# Septic Thrombophlebitis with Persistent Bacteremia Treated by Thrombolysis

**DOI:** 10.5152/TJAR.2021.1125

**Published:** 2022-04-01

**Authors:** Boris Farny, Charles-Hervé Vacheron, Arnaud Friggeri, Bernard Allaouchiche

**Affiliations:** 1 Brûlés Pierre Colson Centre, Edouard Herriot Hospital, Hospice Civil de Lyon, Lyon, France; 2 Claude Bernard Lyon University Faculty of Medicine, Lyon, France; 3 Department of Anaesthesiology and Reanimation, Centre Hospitalier Lyon-Sud, Hospice Civil de Lyon, Lyon, France

**Keywords:** PICC line, septic thrombophlebitis, thrombolysis

## Abstract

Septic thrombophlebitis requires both adapted antimicrobial therapy and control of the source of the infection. The source of the infection is generally controlled through adequate management of anticoagulation. In this study, we present the case of a 50 years old woman, with septic thrombophlebitis of a peripherally inserted central catheters line and with persistent bacteremia and thrombus, where in situ thrombolysis has successfully removed the thrombus.

**Main Points**

- Septic thrombophlebitis is a rare complication of a catheter.
- Treatment relies on adequately managed anticoagulation intervention and adapted antimicrobial therapy.
- In case of persistence of the thrombus, despite the applied anticoagulation regimen, in situ thrombolysis is an option to be able to control the infection.

## Introduction

Septic thrombophlebitis is an uncommon condition with limited data access. This happens commonly after catheter insertion and requires a complex management strategy and is associated with a high rate of mortality. In this study, we report here a case of a 50-year-old female who developed septic thrombophlebitis associated with a peripherally inserted central catheter (PICC) line. Written consent for the publication of the patient case was gathered.

## Case Presentation

A 50 years old woman with a history of type 2 diabetes mellitus, arterial hypertension, hyperthyroidism, intellectual disability, and morbid obesity (body mass index = 53.4 kg m^2^) was diagnosed in October with right-sided breast cancer (poorly differentiated ductal carcinoma). The investigation found an isolated right axillar node with no metastases. In order to perform chemotherapy (docetaxel, epidoxorubicin, and cyclophosphamide), a PICC line was placed into the right basilic vein (*Lifecath PICC, VYGON* *^®^*). The first chemotherapy administration was performed on October, 24.

On December 2 2018 of the same year, the patient presented with acute heart failure from atrial fibrillation with hyperthermia. The PICC line insertion point showed signs of inflammation with a purulent discharge. During the admission, several blood cultures were collected. The patient developed unfortunately rapidly acute respiratory failure, requiring endotracheal intubation and her admission into an intensive care unit on December 3. Blood culture showed *Staphylococcus aureus *infection, and empiric antimicrobial therapy was introduced (vancomycin and gentamicin). On December 4, an antibiogram revealed a methicillin-susceptible *S. aureus, *and the antimicrobial therapy was therefore adapted to oxacillin and gentamicin. The removal of the PICC line was performed on the same day and the culture of the catheter confirmed the catheter-related blood-borne infection with the same bacteria. Echography of the right arm revealed thrombophlebitis starting from the basilic vein to the end of the subclavian vein. No infectious collection was present, and therefore no surgical intervention was planned. Trans-oesophageal echocardiography did not find any sign of endocarditis, and cerebral, thoracic, abdominal, and pelvic computed tomography did not identify any pulmonary or visceral embolisms.

Successful therapeutic anticoagulation was achieved with a continuous infusion of unfractionated heparin. The administration of gentamicin was discontinued after 5 days and the antimicrobial therapy was pursued with oxacillin and clindamycin. Daily blood culture stayed positive with *S. aureus*, leading to the addition of daptomycin on December 15.

The persistence of thrombophlebitis prompted us to perform in situ fibrinolysis. Initiating on December 19, a catheter was introduced into the beginning of the basilic vein, and the injection of a bolus of urokinase (100 000 IU), followed by a continuous infusion (10 000 IU h^−1^) until December 23. There was a small persistence of the thrombus with no indication of thrombectomy, allowing the switch from urokinase to unfractionated heparin to achieve therapeutic anticoagulation. There was no subsequent record of the resurgence of the bacteremia.

The patient was discharged from the intensive care unit on December 31, with antimicrobial therapy and curative anticoagulation for 6 weeks. The Doppler ultrasonography showed the total disappearance of thrombophlebitis by January 15. The last blood culture was performed on January 16, confirming the absence of resurgence of the bacteremia. 

## Discussion

Septic thrombophlebitis requires both adapted antimicrobial treatment and the lysis of the thrombus. For the most part, to stay away from the development of the blood clot and in its normal end, curative anticoagulation (by heparin or oral anticoagulant) is sufficient to dissolve the thrombus. 

We conducted an in situ thrombolysis in a case of septic thrombophlebitis resistant to adapted antimicrobial treatment and adequate curative anticoagulation. In this case, the discussion of the in situ thrombolysis is balanced by the infectious risk of the insertion of a catheter in an infected region. However, the remaining option (systemic thrombolysis or surgical thrombectomy) involves a high risk of intracerebral hemorrhage or postoperative complication. To avoid bloodstream infection related to the catheter of thrombolysis, a careful daily echographic evaluation of the lysis of the thrombus is required and early removal of the catheter needs to be performed. 

A systematic review from Cochrane in 2014 highlighted the possibility of thrombolysis compared to anticoagulation in order to lower the incidence of post-thrombotic syndrome.^1^ The opinions expressed in the literature remain heterogeneous without clear guidelines, in regards to thrombolysis treatment choices, measurements, and courses of organization.

In our case, there was a resistance of thrombus to anticoagulation, leading us to choose in situ thrombolysis. A case report by Block et al.^2^ showed a similar case of septic thrombophlebitis due to infection with Candida albicans. In this case, the in situ thrombolysis was performed using urokinase, delivered with an infusion rate of 100 000 IU h^−1^ during a period of 24 hours, achieving good results within 48 hours, thereby allowing for the removal of the catheter. Volkow et al.^3^ used another thrombolysis agent (streptokinase) with an infusion rate ranging from 20 000 to 40 000 IU h^−1^, using a bolus at the start of the thrombolysis (250 000 IU). This regimen was found to be successful in the treatment of 3 patients Schifferdecker et al.^4^ also reported better efficacy using in situ thrombolysis compared to general thrombolysis. This treatment regimen provides a combinatorial advantage as it removes both the thrombus and the source of infection while also lowering the thrombolysis agent dosage required, thereby reducing the risk of bleeding.

## Conclusion

The control of the septic source remains on 2 points: an antimicrobial therapy, adapted to the germ and for a long duration, and on the removal of the catheter. The catheter can be removed only after the complete dissolution of the thrombus in order to avoid any pulmonary embolism. After the ablation of the catheter, daily blood culture needs to be sampled in order to ensure that the bacteremia has been eliminated.

In case of counter indication of thrombolysis, surgical thrombectomy is the last treatment resort, as reported by Krauthamer et al.^5^ in septic thrombophlebitis.
